# Do Global Cities Enable Global Views? Using Twitter to Quantify the Level of Geographical Awareness of U.S. Cities

**DOI:** 10.1371/journal.pone.0132464

**Published:** 2015-07-13

**Authors:** Su Yeon Han, Ming-Hsiang Tsou, Keith C. Clarke

**Affiliations:** 1 Department of Geography, San Diego State University, San Diego, CA, United States of America; 2 Department of Geography, University of California Santa Barbara, Santa Barbara, CA, United States of America; Universidad Veracruzana, MEXICO

## Abstract

Dynamic social media content, such as Twitter messages, can be used to examine individuals’ beliefs and perceptions. By analyzing Twitter messages, this study examines how Twitter users exchanged and recognized toponyms (city names) for different cities in the United States. The frequency and variety of city names found in their online conversations were used to identify the unique spatiotemporal patterns of “geographical awareness” for Twitter users. A new analytic method, Knowledge Discovery in Cyberspace for Geographical Awareness (KDCGA), is introduced to help identify the dynamic spatiotemporal patterns of geographic awareness among social media conversations. Twitter data were collected across 50 U.S. cities. Thousands of city names around the world were extracted from a large volume of Twitter messages (over 5 million tweets) by using the Twitter Application Programming Interface (APIs) and Python language computer programs. The percentages of distant city names (cities located in distant states or other countries far away from the locations of Twitter users) were used to estimate the level of global geographical awareness for Twitter users in each U.S. city. A Global awareness index (GAI) was developed to quantify the level of geographical awareness of Twitter users from within the same city. Our findings are that: (1) the level of geographical awareness varies depending on when and where Twitter messages are posted, yet Twitter users from big cities are more aware of the names of international cities or distant US cities than users from mid-size cities; (2) Twitter users have an increased awareness of other city names far away from their home city during holiday seasons; and (3) Twitter users are more aware of nearby city names than distant city names, and more aware of big city names rather than small city names.

## Introduction

Internet-based human communication and messaging has rapidly expanded due to social networking sites (SNS) where people continue to share their ideas, images, news, memes and advertisements at a prodigious and increasing rate. SNS (such as Twitter, Foursquare, and Flickr) dynamically produce huge quantities of instant messages that often reveal the locations of their users and the locations where the messages were created. The locations of users can be found in their user profiles, if they disclose this information to the public. In some cases, global positioning systems (GPS) in mobile phones are used to identify the latitude and longitude where the messages were created, known as geotags. Social media data with geolocation tags or location profiles are being increasingly used in geographical research projects, and in the emerging business of location based services [1–7]. However, few studies have utilized the actual geographical names (i.e., toponyms or place names) mentioned in social media content to estimate the geographical awareness of particular groups and individuals from the same city.

This research aims to employ the names of cities mentioned in Twitter messages to understand Twitter users’ geographical awareness. A new analytic method, Knowledge Discovery in Cyberspace for Geographical Awareness (KDCGA), has been developed to facilitate the discovery of geographical knowledge from large volumes of social media data. In developing the KDCGA, about 5 million Twitter messages were collected across 50 cities in the U.S. over three months. Thousands of city names throughout the world were identified from the Twitter messages (tweets). The frequency of mentioning of city names was quantified and used as an element to estimate the level of geographical awareness for Twitter users living in the same city. This estimation enables a comparison of the spatial and temporal change of geographical awareness for Twitter users from different U. S. cities over time.

Quantifying the level of geographical awareness is a key element in this study. We introduce the global awareness index (GAI) to quantify the level of geographical awareness of Twitter users from the same city. A high GAI indicates that Twitter users from the same city mention international city names, or distant U.S. city names, much more than nearby local city names. Conversely, a low GAI indicates that the frequency of international or distant city names mentioned in the messages is lower than the frequency of local city names mentioned. In short, Twitter users who mention faraway city names more frequently have a higher GAI—i.e., levels of geographical awareness. We propose that the GAI as developed in this study can be used to quantify the level of globalization worldwide. Many researchers have attempted to quantify the level of globalization in the form of indices and argued the importance of quantification [8–11]. One unique aspect of using the GAI to quantify globalization is the exchange of human messages in cyberspace, so the index can reflect dynamic human behaviors in real time.

One goal of this study is to test Tobler’s first law of geography in cyberspace. The law states: “*Everything is related to everything else*, *but near things are more related than distant things*.” [12]. If Tobler’s first law of geography is applicable in cyberspace, Twitter users’ perceptions and communications should be more related to nearby regions and cities than to regions that are far away. Therefore, they should mention the names of places close to their home more frequently than the names of places that are far away, i.e. New Yorkers should mention Hoboken, New Jersey more often than Santa Barbara, California. However, some scholars have argued for the “death of geography” in the information age [13, 14]–i.e. that the information space of interconnected worldwide networks enables emerging social space in cyberspace connecting individuals beyond the physical boundary of the human body and geographic space; which may result in the “collapse” of space and time. If the meaning of distance disappears in cyberspace, individuals should be equally aware of city names distant from themselves and the city names nearby their home. This study examines this question and explores whether geographic distance still matters in relation to human perceptions of space and place within cyberspace.

The research is intended to address the following three questions:

1. In what cities do Twitter users have high levels of geographical awareness? It is hypothesized that Twitter users in the largest cities (e.g., New York City; Los Angeles) are more aware of distant city names and international city names than those who live in mid-size cities (e.g., Jacksonville, Florida; Portland, Oregon).
2. How does the geographical awareness of Twitter users change over time? What differences in the temporal level of geographical awareness characterize Twitter users from different regions? It is hypothesized that Twitter users tend to mention far-away city names from their residence more frequently during holidays than during regular days.
3. What are the spatial patterns of geographical awareness for Twitter users from the same or different cities? It is hypothesized that Twitter users are likely to mention closer cities than distant cities. However, they might be more likely to be more aware of big city names rather than small city names.

## Background

Social media applications such as Twitter and Facebook have recently gained a spectacular popularity. According to Twitter, over 500 million active Twitter users produced about 350 million messages per day in 2013. To exploit these massive social media data bases, extensive studies have been conducted to develop human knowledge, including analyzing human communication and networks [15–17], sentiment analysis [18–22], homeland security [23–25], predictions of election results [4], predictions of the stock market [26], crisis management [5, 27], and tracking infectious diseases [6, 7, 28]. These research projects have shown the tremendous value of social media analytics. Rapid growth of social networking sites has triggered not only extensive research in academia, but also the development of applications making use of social media data in the private sectors and commercial applications, such as in mobile phone apps and location-aware devices.

### Cognitive geography and social media

Cognitive geography focuses on the study of how the human mind perceives and interacts with its geographical surroundings through the senses. Cognitive geography is based on the idea that individuals apply learned geographic knowledge about the spatial and non-spatial characteristics of their environment. The knowledge is gained, processed and stored in the human mental system [29]. The organized and decipherable representations of this knowledge is referred to as cognitive or mental maps [29]. These maps provide geographical knowledge that allows us to easily analyze and comprehend what individuals perceive in their mind especially in relation to space. It is crucial to understand how people recognize the space around them, as a number of researchers argue that interpreting individual’s perception of space is crucial to explaining human behavior [30]. In this study, Twitter messages were used to represent how individuals recognize nearby and distant places in their mind. Frequent generation of tweets leads to massive data volumes, which can be analyzed to understand individual’s perceptions, emotions, behaviors and actions in relation to the places around them.

Some studies have attempted to evaluate an individual’s level of cognition or awareness about space and places [31, 32]. Recently, Xu *et al*. [33] analyzed Twitter messages to evaluate the geographical awareness of the users. Their study investigated the geographical awareness of a small set of users and identified their different types of geographical awareness. Their study also conducted a qualitative analysis to understand the relationship between each user’s personal information (e.g., jobs) and the level of geographical awareness.

### Definition of geographical awareness

In the current study, the act of naming a place within one’s Twitter messages is assumed to imply awareness of that name. This does not necessarily mean that the Twitter users can pinpoint the exact location of the cities or describe detailed information about the cities. For example, if a user tweets, “I am going to Chicago for the annual meeting this year,” the user already knows or is aware of the city of Chicago. But we cannot tell if users have sufficient geographic knowledge about Chicago, such as the exact location of Chicago or the total population of Chicago. Geographical awareness is different from geographic knowledge. Care must be taken to distinguish among different cities with the same name (Paris, France vs. Paris, Texas) and between dissimilar semantics (London, England vs. Jack London, Writer).

### Exploring big data to define and evaluate geographical awareness

Social media research has increasingly focused on the analysis of big data [21, 34, 35]. Big data is an evolving term. It has been used to refer to data sets that are too big to process with existing methods [36]. Tsou recently described the characteristics of big data while focusing on social media data. He stated that big data are dynamically linked–changing over time and across places, non-structured and in a hybrid format. Since big data are human-centered and human-created, they provides a basis that enables one to explore human behavior. Big data also contains many errors and noise—typically over 60% of the records are useless, but the content can still provide insightful and valuable knowledge for research and businesses [37]. The aggregate of millions of messages has a potential to reflect social phenomena related to particular events [35]. In our study, millions of Twitter messages were collected from various geographical regions across the U.S. This comprehensive collection of Twitter data allowed us to estimate and compare the level of geographical awareness of millions of Twitter users who reside in various cities in the U.S.

## Data Collection

Twitter is an Internet-based social networking and microblogging service that permits registered users to receive and broadcast very short text messages (tweets), up to 140 characters in length. Users and collective sites are identified by user names and hashtag identifiers such as “#katyperry”. Messages can be resent or forwarded (retweeted), and groups of associates can associate together (as friends) in sending and receiving group tweets. References to Internet URLs are made by compression, for example into tinyurls.

To collect and analyze the spatial and temporal patterns of Twitter messages, two steps are involved. The first step is to collect tweets using Twitter Application Programming Interfaces (APIs) [38], which allows a sample of tweets to be queried by multiple user-defined criteria. For example, we can query tweets containing certain hashtags or keywords within a region or identify the most popular retweets (RT) from specific users. There are two types of Twitter APIs, Streaming APIs and REST APIs. This study adopted Twitter Search APIs for our major data collection tools, which is a part of REST APIs. The Twitter Search APIs allow us to query tweets that contain predefined keywords within a defined radius of a location. For example, we can query any tweets containing “Whooping Cough” within a 20 km radius buffer area outward from the center of San Diego.

**Fig 1:**
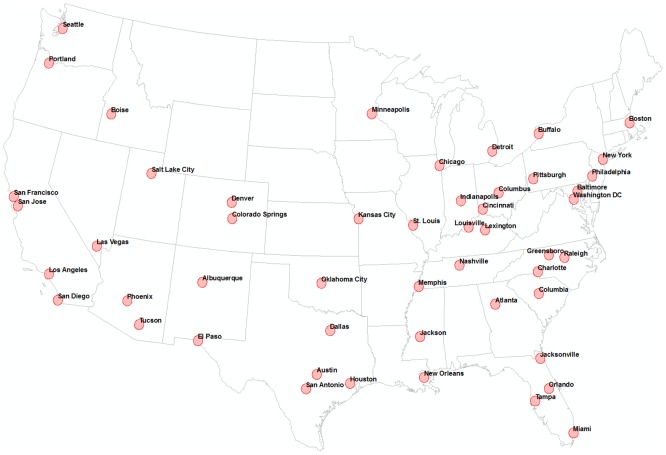
Home cities. Tweets were collected within a 20 mile buffer from each center of the 50 major U.S. cities.

In this study, tweets containing the names of cities all over the world were collected within a 20 mile buffer from the center of each of 50 cities in the U.S. (Fig 1). These cities are the home cities of Twitter users. These 50 home cities were selected across the various geographical regions in the U.S. with most cities in densely populated areas. In each of the 50 home cities, two datasets were collected. The first dataset was tweets that contained the names of cities with populations over 100,000 in the U.S. and the names of cities with population over 500,000 outside of the U.S. The second dataset was tweets that had the names of cities with a population over 10,000 within the U.S. The first dataset was collected between the dates of December 1, 2013 and February 28, 2014 inclusive, and the second dataset was collected between December 10, 2013 and February 10, 2014. Retweets were excluded because they are re-postings of someone else’s content, and not new information. After excluding retweets, the first dataset consisted of approximately 3.5 million tweets and the second dataset approximately 1.5 million tweets (Table 1).

**Table 1:** Two datasets of tweets.

	Dataset 1	Dataset 2
Search areas (using Twitter Search API)	20 mile buffer area from the center of each 50 city represented in the map of Fig 1	20 mile buffer area from the center of each 50 city represented in the map of Fig 1
	Name of large cities in both the U.S. and over the world	Name of cities in the U.S.
The number of population in selected city names	Cities with population over 100,000 in the U.S. and 500,000 outside the U.S.	Cities with population over 10,000 in the U.S.
The total number of cities	1,860	3,882
Data collection period	12/1/2013 to 2/28/2014	12/10/2013 to 2/10/2014
The total number of tweets including keywords (cities names)	3,515,887 tweets	1,497,721 tweets

Every tweet in the first dataset includes at least one name of a large city in the U.S. or elsewhere. The second dataset does not include city names outside the U.S., but contains the names of small, mid-sized, and large cities in the U.S.

For geocoding processes, place name gazetteers were provided by the GeoNet Names server, supported by the National Geospatial Intelligence Agency (earth-info.nga.mil/gns/html). This geographical gazetteer database covers all countries and contains over eight million place names in multiple languages. This gazetteer is useful for geocoding since it provides the coordinates of all cities in the world. The challenges in geocoding are discussed in detail in the methodology section below.

## Data Analysis and Visualization

Focusing on big data analysis of social media messages, this study adopted the research framework of Knowledge Discovery in Cyberspace (KDC) [39]. KDC is particularly designed for knowledge discovery in social media and big data, focusing on the interdependent relationship among place, time, and content. A new aspect of KDC is to discover the dynamic spatiotemporal patterns of massive amounts of social media messages by using highly scalable information mining algorithms, geographic information systems (GIS), visualization tools, and spatial statistical methods.

**Fig 2:**
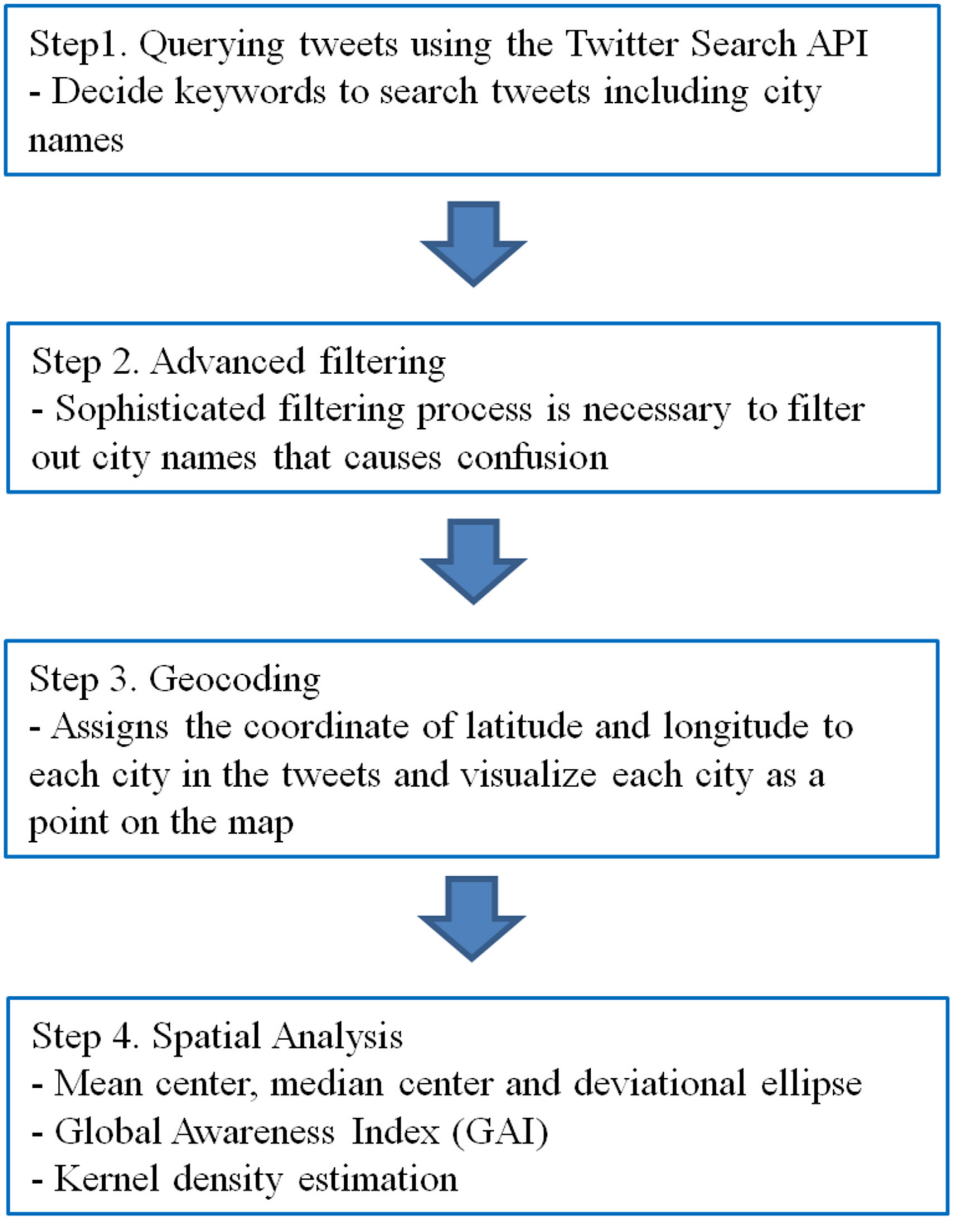
Four steps of knowledge discovery in cyberspace for geographical awareness (KDCGA). The first and second steps select tweets containing the city names. The third step is to locate and visualize the tweets on the map. The fourth step is to reveal spatiotemporal patterns by using spatial statistical methods.

Based on the original framework of KDC, this study introduced a new method, called Knowledge Discovery in Cyberspace for Geographical Awareness (KDCGA) (Fig 2). KDCGA is designed specifically for discovering users’ geographical awareness by analyzing their social media messages. KDCGA can be used to examine the interdependent relationships among place, time and messages (such as users’ geographical awareness). For example, the estimated geographical awareness from Twitter users in Los Angeles (LA) is different from the estimated geographical awareness from users in Charlotte, North Carolina. Our hypothesis is that users from different cities have different levels of geographical awareness. In addition, the level of geographical awareness can change over time. For example, individuals might have higher global geographical awareness around major holidays such as the Christmas and New Year Holidays because many individuals are likely to have travel plans during the holidays, or be sending gifts and messages. KDCGA consists of four procedures to estimate the level of geographical awareness (Fig 2).

The first step of KDCGA is to query tweets using the Twitter Search APIs with keywords being the list of city names. Twitter Search APIs provide their own query functions that allow us to query tweets including defined keywords. The original Twitter data has many elements such as the text of the messages, screen name, profile background url, followers count, friends count, and hashtag. When the query function provided by Twitter Search API is used, the program queries tweets containing the city names anywhere in all of these elements. Therefore, above all, the program removes tweets that do not contain the keywords (city names) in the text of the Twitter messages. After this process, only those tweets that contain the keywords in the text remain.

A major procedure in the first step is to define the keywords (city names) for collecting tweets. A problem in deciding keywords is that many cities in the world share the same name, and that some place names are words in another context. Table 2 shows a few cities that have the same name and are located in at least two different regions. Clarke notes that 26 different places worldwide share the placename “Paris” [40].

**Table 2:** Examples of cities that share the same name and are located in different regions.

City Name	States in the U.S. or Countries Where the City is Located	States in the U.S. or Countries Where the City is Located
1. Richmond	Texas	Virginia
2. Gainesville	Florida	Virginia
3. Charleston	Washington	South Carolina
4. Memphis	Tennessee	Kentucky
5. Athens	Greece	Georgia
6. Paris	France	Texas

Richmond, Gainesville, and Charleston have the same name but are located in different states in the U.S. Athens and Paris have the same name but are located in different countries.

To analyze distributional patterns of tweets containing city names, these tweets are supposed to be geocoded in the later step. In terms of geocoding, if all city names in the world were unique, it would be straightforward to match each city name contained in the tweets with the coordinate of each city provided by the gazetteer. However, in reality, there are two or more different cities that have identical names (Table 2), which can cause confusion in searching for tweets containing these names. For example, if the first Twitter message says “I am going to visit the historical place, Acropolis of Athens” and the second Twitter message says “It takes only about one hour from Atlanta to Athens”, a human can recognize that the first user is talking about Athens in Greece, and the second user about Athens in Georgia. However, current technology cannot distinguish which Athens the user means.

To prevent confusion, our program only queries tweets containing both names of cities and names of the states or country that the cities belong to. This has the advantage of also eliminating the non-toponyms, such as Jack London. Table 3 shows the rule for deciding the format of keywords. Another reason to use this rule is not to overestimate or underestimate the number of tweets that include the set of city names. If we collect tweets that contain only “Los Angeles” without “California”, or “CA” on the one hand and collect tweets that contain “Athens” with “Georgia” or “GR” on the other hand, the number of tweets containing the city name of Los Angeles would be overestimated compared to the number of tweets containing Athens, Georgia or GR. From this reason, the same rule to decide the keywords was applied for both the cities with the same name in different places (e.g., 1st to 6th keywords in Table 3), and the cities that have a unique name (e.g., 7th and 8th keywords in Table 3).

**Table 3:** Examples of keywords by using combined query functions in Twitter Search APIs.

The Rule of Deciding the Format of Keywords (Row), City Name (Column)	City Name and State Name or Country Name	City Name and State Name or Country Name
	City Name and State Code or Country Code	City Name and State Code or Country Code
1. Richmond	Richmond Texas	Richmond Virginia
	Richmond TX	Richmond VA
2. Gainesville	Gainesville Florida	Gainesville Virginia
	Gainesville FL	Gainesville VA
3. Charleston	Charleston Washington	Charleston South Carolina
	Charleston WA	Charleston SC
4. Memphis	Memphis Tennessee	Memphis Kentucky
	Memphis TN	Memphis KY
5. Athens	Athens Georgia	Athens Greece
	Athens GA	Athens GR
6. Paris	Paris France	Paris Texas
	Paris FR	Paris TX
7. Los Angeles	Los Angeles California	Not applicable
	Los Angeles CA	Not applicable
8. Tokyo	Tokyo Japan	Not applicable
	Tokyo JP	Not applicable

#1–#6 are the examples of keywords for different cities with the same name. #7 and #8 are the examples of keywords for cities that have unique names. City names without country names or state names are not chosen because identical city names cause confusion in geocoding them.

The second step is a process of advanced filtering. This process aims to eliminate the tweets that do not contain actual city names as much as possible. The keywords used in the Twitter search APIs need to follow predefined Boolean operations and query string formats. Some city names as keywords can create unexpected query results. For example, if we search tweets with the keyword, “Athens, Georgia”, the Twitter query function would search any tweets containing the two individual keywords: “Athens”, and “Georgia”. In other words, the query function selects all tweets containing those two words anywhere in the twitter data regardless of whether they are uppercase or lowercase, together or separated. One possible tweet of an unexpected query result that does not contain an actual city name could be “Now the Korean drama, the battle of athens, is available at Zion market, Georgia” This tweet contain the city name, “athens” and state name, “Georgia”, but the words in the tweet actually do not mean the city of Athens in the state of Georgia. In the advanced filtering process, the program removes the tweets that do not contain a city name and a state name next to each other or a city name and a country name or code next to each other if the city is the outside the U.S. As long as these words are positioned together, the city names are valid regardless of whether they are uppercase or lowercase. For example, city names in tweets such as “San Diego, California”, “California san diego”, “Athens of Greece” and “Athens in Georgia” are acceptable.

The last two steps are for the visualization and spatial analysis of the tweets collected in the previous steps. The third step is to match each city name mentioned in the tweet with the geographic coordinates of the city, and to visualize them as points on the map. The coordinates of each city are available from gazetteers provided by the GeoNames gazetteer. If city names were mentioned multiple times in a single tweet, each city name was geocoded and represented as a dot on the map.

Step four consists of a set of various spatial analyses to estimate the level of geographical awareness. To examine the difference of spatial distribution of city names mentioned in the tweets in different regions, three centrographic measures–mean center, median center and standard deviational ellipse—were adopted from previous work [33]. The mean center is the average of the *x* and *y* coordinates of all points, representing the overall location of points. The mean center is influenced by outliers far away from the major clusters, and must be converted from spherical to linear distance. The median center is another way to represent the overall location of points by identifying the location that minimizes overall Euclidean distance to the points in a dataset. The median center is less influenced by outliers than the mean center. The standard deviational ellipse summarizes the spatial characteristic of a set of geographic features and provides metrics of central tendency, dispersion and directional trend [41].

The global awareness index (GAI) was created to compare the differences in the levels of geographical awareness of Twitter users living in different regions. In this study, GAI at each of the 50 cities was measured and compared. The mathematical expression for calculating GAI can be given as:

$$Global\;Awareness\;Index\;(GAI)\;=\;\frac{1}{p}\sum_{i=1}^{n}Di\;*\;Xi$$ (1)

where *n* is the total number of keywords (cities mentioned in tweets) at one of the 50 home cities in Fig 1. *Xi* is the number of tweets containing a particular keyword (city name). *Di* is a dimensionless value to indicate the distance between the center of one of home cities and the center of a city mentioned in tweets. To calculate *Di*, the actual distance is calculated and divided by the half length of earth’s circumference (the maximum possible city separation). Great-circle distance was used to calculate the distance between the two places on the globe. *p* is the number of people resident in each of the 50 home cities according to the U.S Census Bureau. Dividing by *p* facilitates a fair comparison of the level of geographical awareness of Twitter users in the different 50 home cities.

The global awareness index (GAI) changes based on the distance, the size of home city, and the number of city names mentioned in tweets. GAI increases as the distance between a home city and the cities mentioned in tweets increases. GAI also increases as the number of cities mentioned in tweets increase. However, GAI decrease as the size of home city increases. A high GAI means that users frequently mention cities that are far away from their residence compared to nearby cities. If Twitter users in region A mention more distant cities than users in region B and the number of population is the same in both cities, region A will have a higher GAI than region B.

Kernel density estimation (KDE) is used to identify the hotspots of tweets. KDE has been used to map the distribution of social media data in many studies—e.g., [2, 4, 21, 34, 35, 42, 43]. KDE inputs *x*, *y*, *z* and outputs a map in raster format where each cell has a value representing the level of intensity. Concentrations of intense values are hotspots. In this study, per Twitter users living in each home city (Fig 1), KDE inputs latitude and longitude of the cities mentioned, and the number of cities at each point, and outputs a map in raster format. To compare the difference of KDE outputs among two groups of Twitter users in different home city, a raster-based map algebra was used to calculate the difference between two KDE maps [4]. The formula is given as:

$$\begin{array}{l} \;Differential\;Map=\;\left(Each\;Cell\;Value\;of\;Map\;A/Maximum\;Cell\;Value\;of\;Map\;A\right)\\ \;-(Each\;Cell\;Value\;of\;Map\;B/Maximum\;Cell\;Value\;of\;Map\;B)\; \end{array}$$ (2)

Where map A is an output in raster format of KDE whose inputs are *x*, *y* (coordinates of the cities mentioned) and z (the frequency of each city mentioned) at one of home cities in Fig 1. Map B is an output in raster format of KDE whose inputs are *x*, *y*, *z* values of tweets created by Twitter users in another home city. This method was used to compare the difference of the level of geographical awareness of Twitter users between pairs of home cities.

## Findings and Interpretation

### The spatial difference of the global geographical awareness of major U.S. cities

**Fig 3:**
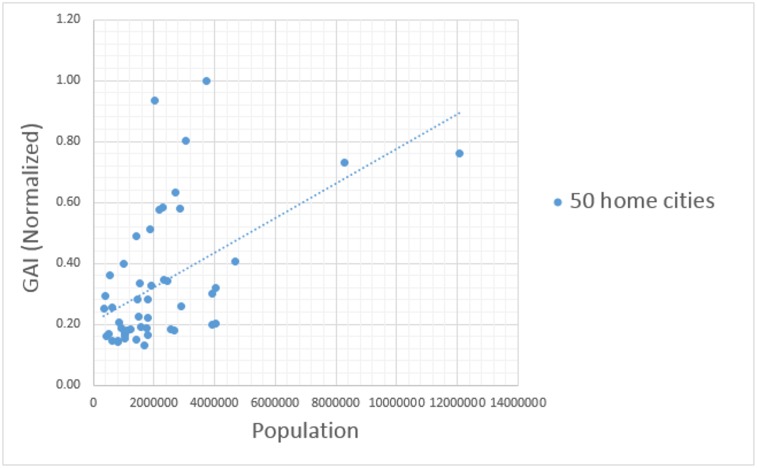
The relationship between population size and global awareness index (GAI). There is a weak positive relationship (Pearson correlation coefficient (*r* = 0.52) between the population within a 20 mile buffer of each of the 50 cities in Fig 1 and the level of geographical awareness of Twitter users in their respective city (S1 Table).

The level of geographical awareness of Twitter users varies depending on where the messages were created. The GAI quantifies the level of geographical awareness and enables comparison of the level of geographical awareness of Twitter users living in different cities in the U.S. Table 4 shows the GAI of Twitter users for each of the 50 home cities (Fig 1) in the U.S. These GAIs are calculated based on dataset 1 in Table 1. Twitter users living in densely populated areas tend to be more aware of international cities or distant U.S. cities than those in less populated areas. A scatter plot with a regression line (Fig 3) shows the relationship between the population within the 20 mile buffer of each city (*x* axis) and the global awareness index (GAI) of Twitter users (*y* axis). The Pearson correlation coefficient (*r*) was used to reveal this relationship because it measures the strength and direction of the linear relationship. The correlation coefficient between the two variables is 0.52, which means that there is a weak positive relationship between the two variables. In other words, the level of geographical awareness (i.e. GAI) is likely to be high in cities with high populations. Also the top 10 highest population cities mostly show fairly high GAIs. Therefore, the general trend is that users living in highly urbanized areas tend to mention far away cities rather than nearby cities in their tweets compared to those in less urbanized areas. This trend implies that big cities have more frequent movement of people, ideas, and commodities between faraway cities than small cities.

**Table 4:** Global Awareness Index (GAI) at 50 cities in the U.S.

a) home city	b) state	d) pop	e) pop rank	f) tweets	g) non US	h) GAI	i) GAI (normalized)
Washington	DC	601723	23	156587	15.09	4.624	1
San Jose*	CA	945942	10	53625	19.66	4.313	0.9327
San Francisco	CA	805235	13	146889	7.86	3.719	0.8042
New York*	NY	8175133	1	355446	16.44	3.517	0.7605
Los Angeles*	CA	3792621	2	247036	12.12	3.383	0.7317
Atlanta	GA	420003	33	157749	5.22	2.927	0.6329
Seattle	WA	608660	22	97459	6.84	2.704	0.5848
Boston	MA	617594	21	95951	10.25	2.683	0.5802
San Diego*	CA	1,307,402	8	59814	12.91	2.665	0.5763
Las Vegas	NV	583756	28	76918	6.84	2.366	0.5117
Austin	TX	790390	14	86277	4.77	2.254	0.4875
Chicago*	IL	2695598	3	170219	6.58	1.883	0.4073
Nashville	TN	601222	24	50511	3.69	1.843	0.3985
Raleigh	NC	403892	35	36835	2.71	1.66	0.359
Dallas*	TX	1197816	9	129608	3.15	1.597	0.3454
Denver	CO	600158	25	125092	4.48	1.575	0.3406
Pittsburgh	PA	305704	41	38794	8.42	1.55	0.3353
Portland	CA	583776	27	71209	2.49	1.509	0.3264
Miami	FL	399457	36	66064	10.13	1.472	0.3184
Houston*	TX	2100263	4	146367	4.21	1.386	0.2998
Boise	ID	205671	47	13572	2.63	1.35	0.2919
Orlando	FL	238300	46	72087	3.3	1.309	0.2831
Charlotte	NC	731424	16	54318	4.19	1.3	0.2811
Tampa	FL	335709	39	68302	5.24	1.188	0.2568
Colorado Springs	CO	416427	34	18722	5.41	1.184	0.2561
Greensboro	NC	269666	44	13365	4.41	1.167	0.2523
Indianapolis	IN	820445	12	39833	4.36	1.039	0.2247
Baltimore	MD	620961	20	49024	4.85	1.02	0.2206
New Orleans	LA	343829	38	20025	4.59	0.947	0.2047
Phoenix*	AZ	1445632	6	121480	3.2	0.927	0.2005
Philadelphia*	PA	1526006	5	69559	6.13	0.909	0.1965
Kansas City	MO	459787	32	105049	1.38	0.889	0.1923
St. Louis	MO	319294	40	27129	6.56	0.865	0.1871
Tucson	AZ	520116	31	21438	4.4	0.858	0.1856
Detroit	MI	713777	17	49530	6.21	0.856	0.1851
Salt Lake City	UT	186440	48	16672	7.14	0.841	0.1818
Minneapolis	MN	382578	37	50540	5.95	0.826	0.1787
Jacksonville	FL	821784	11	40039	2.54	0.825	0.1785
Buffalo	NY	261310	45	18903	6.32	0.802	0.1734
Lexington	KY	295803	43	17311	2.5	0.786	0.17
Oklahoma City	OK	579999	29	30987	3.43	0.782	0.1692
San Antonio*	TX	1327407	7	43803	4.49	0.755	0.1632
Memphis	TN	646889	19	32573	2.84	0.755	0.1632
Jackson	MS	173514	49	8041	5.38	0.741	0.1603
Louisville	KY	597337	26	30590	2.5	0.7	0.1514
Columbus	OH	787033	15	37772	3.09	0.685	0.1482
Columbia	SC	129272	50	13448	3.51	0.678	0.1466
Albuquerque	NM	545852	30	16164	4.4	0.667	0.1443
El Paso	TX	649121	18	18629	2.61	0.656	0.142
Cincinnati	IN	296943	42	28532	3.94	0.601	0.1299

Column D shows the population in each city based on the 2010 census. Column E indicates the rank of population in each city. Column F represents the number of tweets collected in each of the 50 home cities in Fig 1. Column G shows the number of tweets containing city names outside the U.S. divided by the total number of tweets. Column H is GAI multiply by 100000. Column I is normalized GAI that ranges between 0 and 1.

* represents the biggest top 10 cities by population.

**Fig 4:**
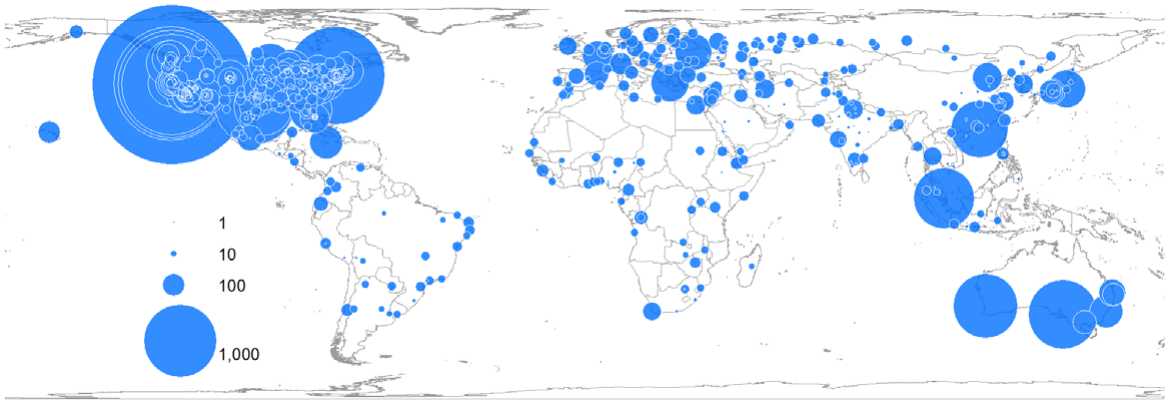
Geographical awareness of Twitter users in San Jose, CA. The size of the circle is proportional to the number of city names mentioned in tweets. Twitter users in San Jose, CA mentioned city names 53,625 times from Dec 2013 to Feb 2014. Among them, the users mentioned San Jose 14,272 times, which is 27% of all the city names mentioned in the tweets. The city name, San Jose, was excluded in this map. The top three most mentioned city names were San Jose, CA, Sunnyvale, CA and San Francisco, CA. This map was created using tweets collected from San Jose, CA during the collection of dataset 1 in Table 1(S2 Table).

**Fig 5:**
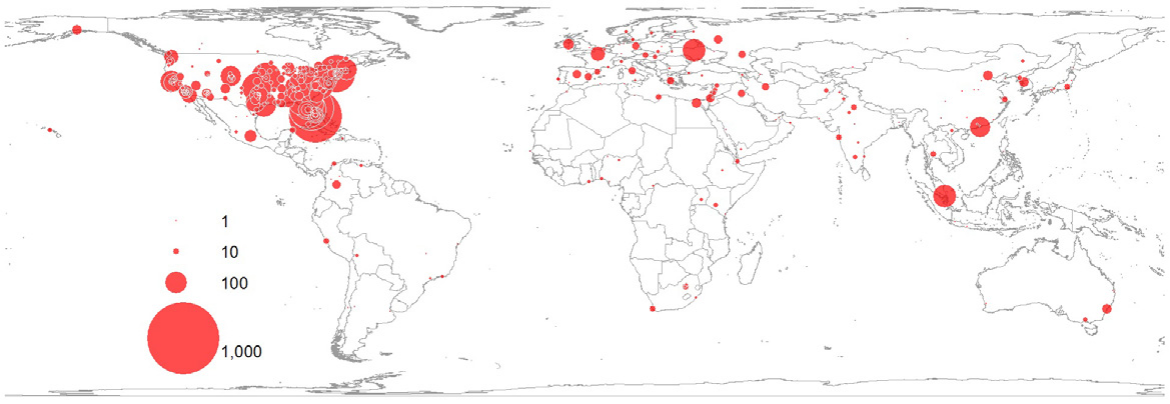
Geographical awareness of Twitter users in Jacksonville, FL. The size of the circle is proportional to the number of city names mentioned in tweets. Twitter users in Jacksonville, FL mentioned city names 40,039 times from Dec 2013 to Feb 2014. Among them, the users mentioned Jacksonville 33,617 times, which is 84% of the entire city names mentioned in the tweets. The city name, Jacksonville, was excluded in this map. The top three most mentioned city names are Jacksonville, FL, Miami, FL, and Orlando, FL. This map was created using tweets collected from Jacksonville, FL during the collection of dataset 1 in Table 1 (S3 Table).

The spatial difference of the geographical awareness can be examined by mapping and using spatial analyses. GAI reveals the arithmetic difference in the geographical awareness of Twitter users living in different cities. On the other hand, mapping and spatial analyses enable the visualization of the distributional patterns of the geographical awareness. For example, in Table 4, San Jose, CA shows a high GAI (0.93), which implies that Twitter users in San Jose recognize distant cities such as cities belonging to far away states in U.S. and global cities, perhaps reflecting the nature of the high-tech workforce there. On the other hand, Jacksonville shows a low GAI (0.18), which implies that Twitter users in this city are not so aware of international cities or distant U.S. cities. Some of the Tweets collected in dataset 1 (Table 1) are mapped in Figs 4 and 5. They show the difference in the distributional patterns of the geographical awareness of Twitter users living in San Jose, CA and Jacksonville, FL. The size of the circles is proportional to the number of city names mentioned in the tweets. Cities with big circles are those of which Twitter users are most aware. Cities with small circles are those of which Twitter users are less aware. More and bigger points are scattered all over the world on the map of San Jose, CA than the map of Jacksonville, FL. Twitter users in San Jose, CA show the higher geographical awareness of cities in every continent than those in Jacksonville, FL. In addition, the users in San Jose are well aware of almost everywhere in the U.S. except the sparely inhabited areas such as deserts and high mountain regions. On the other hand, Twitter users in Jacksonville, FL are well aware of cities in the southeastern U.S., but they show relatively low geographical awareness of cities in western, central and northern U.S.

**Fig 6:**
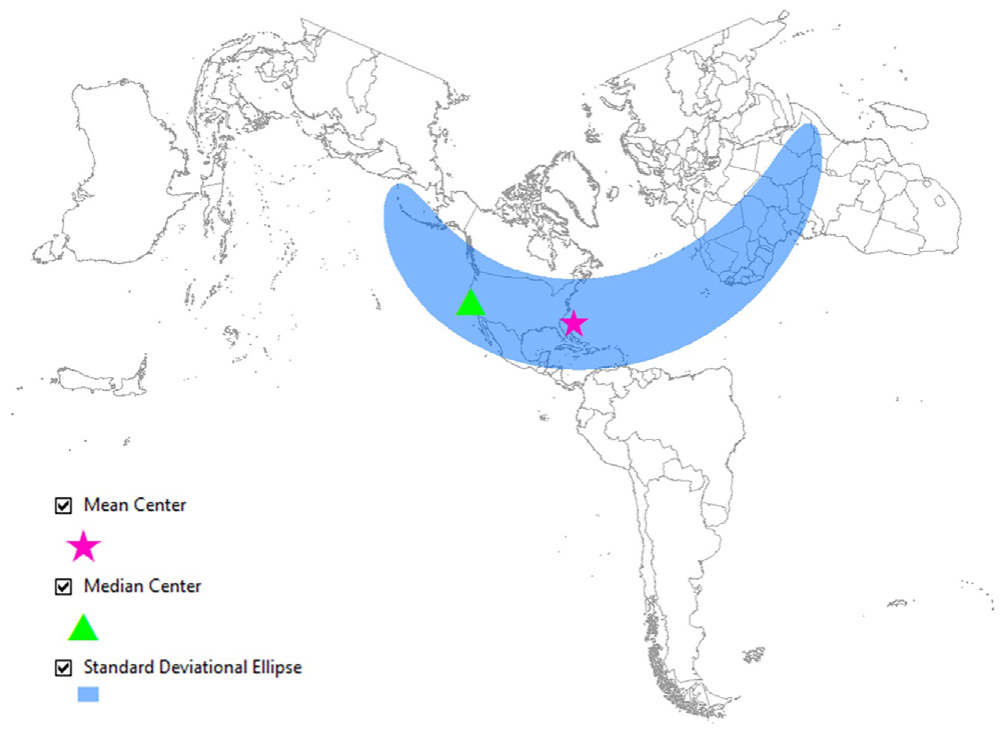
Twitter users with national and international levels of awareness from San Jose, CA. The map shows the central tendency, dispersion and directional trends of the tweets mapped in Fig 4. The widely stretched ellipse shows that the users are well aware of cities belonging to faraway states and international cities. Map projection: Lambert Conformal Conic. (S2 Table)

**Fig 7:**
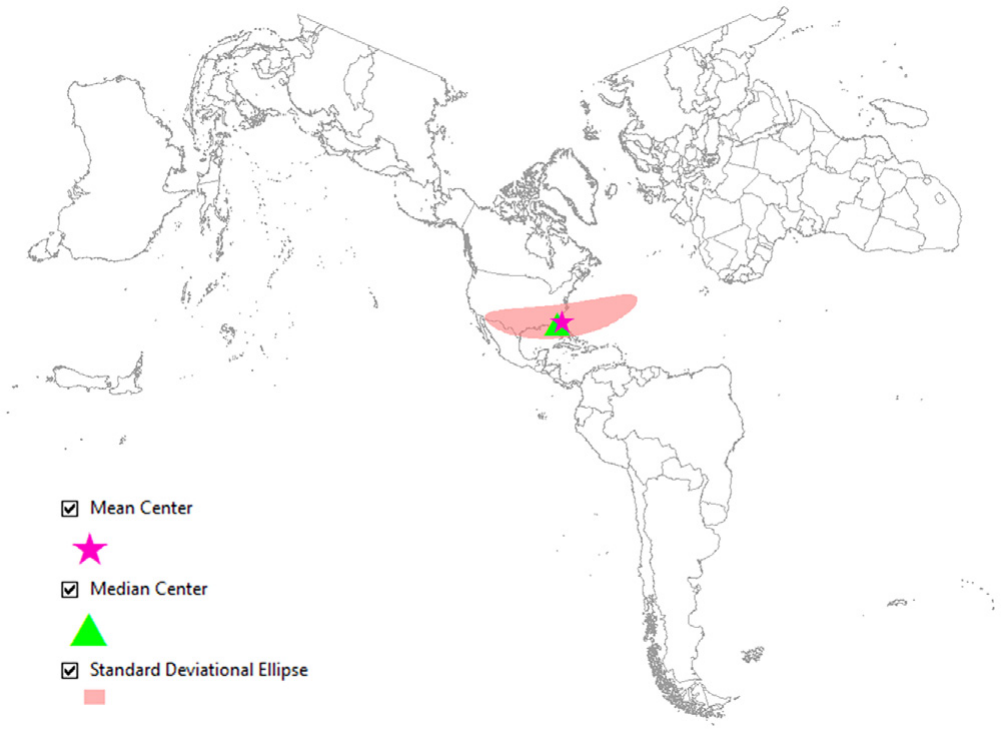
Twitter users with regional and local levels of awareness from Jacksonville, FL. The map shows the central tendency, dispersion and directional trends of the tweets mapped in Fig 5. The narrow ellipse shows that the users are more aware of regional and local cities rather than international cities. Map projection: Lambert Conformal Conic. (S3 Table)

Figs 6 and 7 represent another visualization of the spatial difference in the geographical awareness between two groups of Twitter users in San Jose, CA and Jacksonville, FL. Figs 6 and 7 show the mean center, the median center and the standard deviational ellipse. These three measurements are calculated based on the points in Figs 4 and 5. Fig 6 shows that the mean center and the median center are far apart from each other–the median center is placed in the local area (San Jose, CA), but the mean center is dragged away from the local area by outliers. These two centers that are positioned far apart show that Twitter users in San Jose frequently mention distant cities such as cities in faraway states and on other continents. Conversely, in Fig 7, both the mean center and the median center are positioned together close to the local area (Jacksonville, FL). The closely located mean and median centers indicate that Twitter users in this city mostly mention cities that are closer to their home; therefore, they are not well aware of distant cities such as cities in western and northern U.S. and international cities. The shape of the standard deviational ellipse also represents the distributional difference in the level of geographical awareness between these two groups. The ellipse in Fig 6 is stretched from northwest to southeast across the Atlantic Ocean because the tweets mentioning city names are dispersed all over the world. This widely stretched ellipse shows that Twitter users in San Jose are well aware of cities on the other side of the Atlantic. Conversely, the small sized ellipse in Fig 7 is because Twitter users in Jacksonville, FL mostly mention local cities rather than faraway U.S. cities and international cities. Therefore, this narrow ellipse indicates the low level of geographical awareness of Twitter users in Jacksonville, FL.

**Fig 8:**
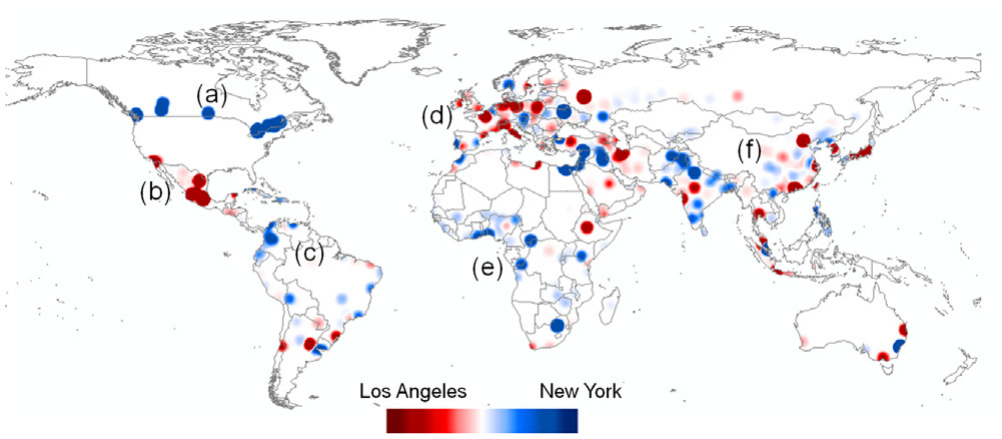
The awareness of global cities between Twitter users in New York (NY) versus Los Angeles (LA). The geographical awareness of each group was estimated based on the names of international cities mentioned in their tweets. The map shows the difference in the distributional patterns of the geographical awareness between the two groups. The users in LA are more aware of the red regions than those in NY. The users in NY are more aware of the blue regions than those in LA. This map was created by using tweets collected from LA and NY during the collection of dataset 1 in Table 1 (S4 and S5 Tables). Tweets inside the U.S. are excluded to map.

There is a clear distributional difference in the awareness of global cities between Twitter users living in New York (NY) and those in Los Angeles (LA). NY and LA were chosen for the comparison because these cities are highly populated and their GAIs are similar—the GAI in NY is 0.76, and in LA is 0.73 (Table 4). First, two maps, one map for NY and the other map for LA, were created using kernel density estimation (KDE). Then, by using Eq 2, the difference between these two KDE maps was calculated and visualized on a red-white-blue color spectrum. Fig 8 shows the difference in distributional patterns of the level of geographical awareness between these two groups, and also reveals the degree of difference in these two groups’ geographical awareness. The dark color represents a big difference, and light color represents a small. Twitter Users in NY are more aware of the blue regions than those in LA and users in LA are more aware of the red regions than those in NY. The users in NY are more aware of Canadian cities (a) than those in LA. Conversely, the users in LA are more aware of Mexican cities (b) than those in NY, possibly because of proximity. The users in NY are more aware of cities located in South America (c) than those in LA, with some exceptions. The users in NY are also more aware of cities in in West and South Africa (e), with the exception of Cape Town. Blue and red areas are mixed in Europe (d) and in Asia (f), while LA dominates Japan, Korea, Taiwan and coastal China. There is a clear pattern in India, with New York favored other than in the tech industry heavy Mumbai and Nagpur.

### Temporal change in the level of geographical awareness

**Fig 9:**
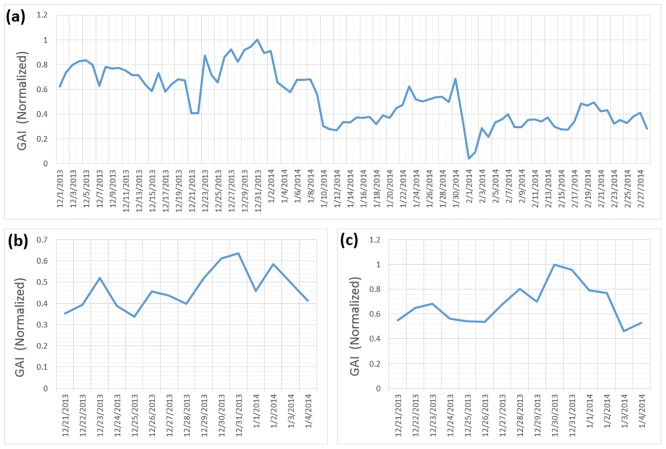
The temporal change of global awareness index (GAI) of Twitter users inU.S. (a) shows the temporal change of the level of geographical awareness of Twitter uesers living in all 50 home cities (Fig 1) from December 2013 to February 2014. The temporal change of the level of geographical awareness is also examined at the city level, in New York City (b) and in Houston, TX (c) around the last week of December. The geographical awareness of the Twitter users was highest in late December 2013.

The level of geographical awareness of Twitter users in the U.S. tends to be high at the end of the calendar year. Fig 9(a) shows the temporal change of the GAI of Twitter users living in all 50 cities (Fig 1). In other words, Fig 9(a) shows the daily change in the level of geographical awareness of users living in major cities of U.S. The GAI starts to increase a few days before December 25th, and reached its peak at the end of 2013. After Dec 31st, GAI droped rapidly, with a low in early February 2014. GAI is mostly high during December, particularly around the last week of December, when compared to January and February. The general trend is that Twitter users in the U.S. are increasingly more aware of distant cities during the holiday season at the end of year, and after the holiday season, users are less aware of distant cities. The temporal changes in the GAI of two groups of Twitter users are compared in Fig 9(b) (New York City) and Fig 9c (Los Angeles) during the end of December and early January. The trend is similar in both regions which show that the level of geographical awareness reaches its peak on December 31st.

### Spatial differences in the level of geographical awareness in the U.S.

**Fig 10:**
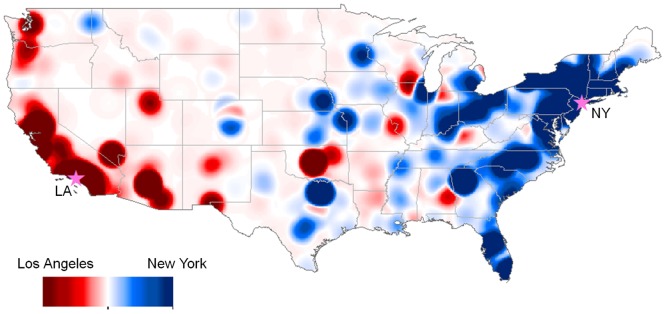
The awareness of U.S. cities between Twitter users in New York (NY) versus Los Angeles (LA). The geographical awareness of each group was estimated based on the names of U.S. cities mentioned in their tweets. The map shows the difference in the distributional patterns of the geographical awareness between the two groups. The users in LA are more aware of the red regions (mostly the western U.S.) than those in NY. The users in NY are more aware of the blue regions (mostly the eastern U.S.) than those in LA. This map was created by using tweets collected from LA and NY during the collection of dataset 2 in Table 1 (S6 and S7 Tables).

Twitter users are more aware of cities that are close to their home than more distant cities in accordance with Tobler’s First Law of Geography. Fig 10 shows the obvious difference in the level of geographical awareness of Twitter users between Twitter users in Los Angeles (LA) and New York City (NY). Twitter users in LA are more aware of the red areas than those in NY. Likewise, Twitter users in New York are more aware of the blue areas than those in LA. The darker the color, the bigger the difference in the level of geographical awareness between the two groups. Users in LA are dominantly aware of cities in the western U.S. and likewise, users in NY are dominantly aware of cities in the eastern U.S, with minor exceptions such as Montgomery, AL and Spokane, WA. The mixed areas colored by light red and light blue in the mid-west indicate that Twitter users in NY and LA are almost equally aware of these regions.

**Fig 11:**
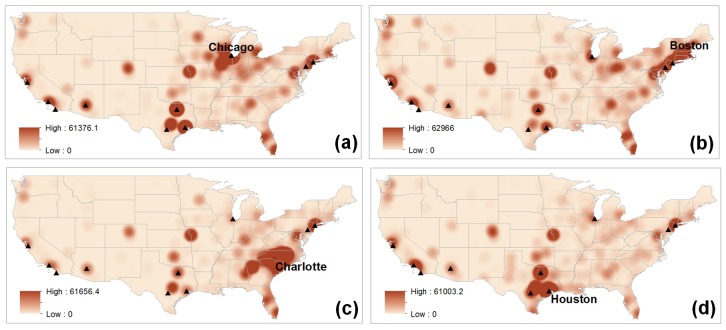
Geographical awareness of Twitter users in Chicago (a), Boston (b), Charlotte (c), and Houston (d). Twitter users are highly aware of hotspots (represented by high intensity), and rarely aware of the regions represented by low intensity. The black triangles represent the top 10 most populated cities. These maps are created using tweets collected from Chicago, Boston, Charlotte and Houston during the collection of dataset 2 in Table 1 (S8, S9, S10 and S11 Tables).

Twitter users are generally more aware of nearby cities than distant cities. However, if cities mentioned in tweets are far away from the city where they live, they are more aware of big cities rather than small cities. Fig 11 shows a hotspot of city names mentioned in tweets that are collected from Chicago (a), Boston (b), Charlotte (c), and Houston (d). In each of the four regions, areas that are close to their residence show a hotspot where users are more aware of nearby cities than cities that are far away from their residence. On the other hand, every map shows a big or small hotspot for the top 10 most populated cities (represented by triangles), which shows that the impact of the distance is less important with big cities than small cities. For example, in Fig 11(b), users in Boston are highly aware of some big cities on the west coast such as Los Angeles and San Francisco even though there is a great distance between these two cities and Boston.

## Conclusion and Discussion

This research analyzed Twitter messages to identify spatiotemporal differences in the level of geographical awareness of Twitter users living in various regions across the U.S. KDCGA, consisting of four steps (querying tweets, filtering, geocoding and spatial analysis) led to the discovery of the following new facts. First, Twitter users living in heavily populated areas have more geographical awareness of cities that are far away than those living in less populated areas. This is an indication of a hierarchical effect in global awareness, the so-called global or world cities have far longer reach than other cities. Second, twitter users are more aware of more distant cities during the holiday season in late December than the remainder of the year. This maybe because of travel planning, gift giving or just staying in touch with distant family and friends. It is possible that other holiday events such as the Muslim Haj, the Chinese lunar new year celebration and the U.S. Spring break show similar patterns. Third, Twitter users are much more aware of nearby cities than distant cities, as predicted by Tobler’s first law of geography. This leads us to conclude that the first law of geography applies equally to both fixed geographic space and the cyberspace of internet-based social media. However, the distance decay of Tobler’s law appears to apply only at one level of the urban hierarchy, such that the largest distance major cities can appear to be virtually closer than smaller nearby cities. Thus to a New Yorker, London England may seem closer than Albany, the state capital just 250km away.

The findings in this study show an effect of distance decay in terms of recognizing city names of Twitter users. Distance decay describes the decline of human interactions between places with increasing distance from their point of origin [44]. The effect of distance decay can be found in daily life. For example, individuals have more telephone calls or mail deliveries between nearby regions than distant regions [44]. Likewise, individuals have more spatial interactions such as visiting friends, traveling and shopping in nearby places than distant places. As a result, Twitter users mention more nearby city names than distant city names so they are more aware of nearby places than distant places (Figs 4, 5, 6, 7, 8, 10 and 11). These findings imply that individuals are likely to interact more with nearby places than distant places. Individuals across the world are interconnected by worldwide networks and form one borderless community in cyberspace. Some scholars have argued that in cyberspace human interactions are not confined to nearby spaces anymore [13, 14]. Nevertheless, the results of this study imply that human interactions in cyberspace are indeed influenced by the friction of distance and reflect Tobler’s first law. People especially living in small cities mostly interact with people in nearby cities, but they rarely interact with people in distant cities.

The level of geographical awareness is not only based on distance, but also on a model of spatial interaction, the gravity model. The gravity model assumes that the attraction or interaction between two cities is proportional to the product of their sizes and inversely proportional to their separation distance [45]. The shorter the distance between two cities is, and the larger the size of either city or both cities is, the greater the expected number of human interactions between the cities. In terms of the geographical awareness of twitter users, they are likely to be more aware of big cities than small cities, especially if the cities are far away from their place of residence (Figs 3, 4, 5 and 11). Global cities are usually very well recognized by people living everywhere, this may be a feature of awareness and less so of interaction. In this case, the effect of distance decay matters less with big cities than small cities. Since there is more attraction between big and nearby cities, big and nearby cities interact more frequently than small and distant cities.

The pattern of geographical awareness of Twitter users demonstrates a particular pattern of spreading ideas called hierarchical diffusion. “Hierarchical diffusion refers to the hopscotch spread from larger, more social dominant centers to smaller ones, typically along the national or international transport network” [45, 46]. For example, K-pop music has diffused in a hierarchical pattern, spreading from a few large cities to smaller cities down the urban hierarchy, even though its medium is cyberspace. Likewise, the users’ geographical awareness of city names diffuses in a hierarchical pattern, spreading from a few highly urbanized cities outward to suburban and rural areas (Fig 11). In other words, users recognize a few big cities, especially the top ten most populated cities (represented by high intensity) and also recognize smaller cities that are close to them (represented by the medium or low intensity). However, faraway small cities are barely recognizable. This phenomenon implies that there is a hierarchical effect in terms of the diffusion of recognizing city names.

The GAI can potentially be used to estimate the relative degree of globalization of cities. Various methods for measuring the level of globalization with a composite index have been discussed—e.g., [8–11]. The GAI created for this study can provide another way of measuring the level of globalization. The GAI quantifies place names that are mentioned in human messages in cyberspace, and could relate to other social media and even traditional information exchanges. The further away and proportionally more the places are mentioned, the larger the GAI is, and the higher the geographical awareness is. GAI is an indicator that can quantitatively show the difference in the level of geographical awareness among groups, such as demographic, cultural or ethnically separate populations. If the individuals in one city have a higher global awareness index (GAI) than those in another city, the former is more likely to be a globalized city than the latter. For example, in cyberspace, if individuals in Los Angeles mention international cities more frequently than those in San Diego, the former has the higher GAI than the latter. The difference in GAI between these two groups can imply that individuals in Los Angeles have more connection to cities belonging to faraway states and in foreign countries than those in San Diego, or the former might be more interested in these faraway cities than the latter due to some reasons such as traveling, working and visiting families and relatives in these cities. In addition, a large volume of human messages mentioning the names of international cities imply that there is potentially active movement of people, ideas, goods, service, labor and capital between the origin city and these international cities. Therefore, in this case, it would make sense to assume that Los Angeles is a more globalized city than San Diego.

The use of social media messages provides a cost effective way to estimate a dynamically changing level of globalization. Globalization indices developed in previous research are computed based on the aggregation of multiple variables revealing cultural, ecological, economic, political and social aspects of countries [47, 48]. They are collected from multiple private and public organizations so that collecting the data is often expensive and time consuming—e.g., [48]. Since it is not trivial to create these data, most indexes of globalization are only measured once a year. However, estimating the level of globalization using social media messages does not require collecting these complex variables, and is essentially instantaneous and continuous. The level and nature of globalization can easily be estimated by collecting place names mentioned in massive user-generated text messages. Individuals are creating the messages that can be used for estimating the level of globalization, and the locations where the messages were created are identifiable. Therefore, by using social media messages, the globalization index can be estimated at local, national, regional, and/or global levels. In addition, real-time and user-generated text messages enable the estimation of level of globalization in any—hourly, daily, monthly and yearly—periods. Since human messages in cyberspace reflect dynamic human activities in real space in real-time, the estimation of the level of globalization based on the human messages can capture the dynamics of the level of globalization.

## Limitations

We acknowledge that publicly available tweets are only a sample of all tweets, and that only a few of them carry a geotag that allows verification that the user is at their stated location, or one of the places mentioned in the text. We further recognize that culling all tweet geography from what is usually transient and fickle information exchanges may reveal more about human intent and interest that actual travel or communication desires. Nevertheless, there is abundant opportunity to learn more about geography from the large numbers of exchanges now enabled by location-aware social media.

## Supporting Information

S1 Table Population size within a 20 mile buffer of each of 50 home cities and the global awareness index (GAI) in their respective city. Column A is each 50 city represented in the map of Fig 1. Column B and C represent coordinate of each city. The number of population within a 20 mile buffer of each 50 cities was estimated based on the census 2010 in Column D. E represents global awareness index (GAI).
(XLSX)

S2 Table The city names and the number of each city name mentioned by Twitter users in San Jose, California. Column A is a list of city names mentioned by Twitter users living in San Jose, California. Column B represents state code or country code where the respective city in column A belongs. Column C represents state name or country name where the respective city in column A belongs. Column D and E represent coordinate of each city. F column shows the population in each city. G column represents the number of tweets mentioned each city.
(XLSX)

S3 Table The city names and the number of each city name mentioned by Twitter users in Jacksonville, Florida. Column A is a list of city names mentioned by Twitter users living in Jacksonville, Florida. Column B represents state code or country code where the respective city in column A belongs. Column C represents state name or country name where the respective city in column A belongs. Column D and E represent coordinate of each city. F column shows the population in each city. G column represents the number of tweets mentioned each city.
(XLSX)

S4 Table The international city names and the number of each international city name mentioned by Twitter users in New York (NY). Column A is a list of international city names mentioned by Twitter users living in NY. Column B represents state code or country code where the respective city in column A belongs. Column C represents state name or country name where the respective city in column A belongs. Column D and E represent coordinate of each city. F column shows the population in each city. G column represents the number of tweets mentioned each city.
(XLSX)

S5 Table The international city names and the number of each international city name mentioned by Twitter users in Los Angeles (LA). Column A is a list of international city names mentioned by Twitter users living in LA. Column B represents state code or country code where the respective city in column A belongs. Column C represents state name or country name where the respective city in column A belongs. Column D and E represent coordinate of each city. F column shows the population in each city. G column represents the number of tweets mentioned each city.
(XLSX)

S6 Table The US city names and the number of each US city name mentioned by Twitter users in New York (NY). Column A is a list of US city names mentioned by Twitter users living in NY. Column B represents state code where the respective city in column A belongs. Column C represents state name where the respective city in column A belongs. Column D and E represent coordinate of each city. F column shows the population in each city. G column represents the number of tweets mentioned each city.
(XLSX)

S7 Table The US city names and the number of each US city name mentioned by Twitter users in Los Angeles (LA). Column A is a list of US city names mentioned by Twitter users living in LA. Column B represents state code where the respective city in column A belongs. Column C represents state name where the respective city in column A belongs. Column D and E represent coordinate of each city. F column shows the population in each city. G column represents the number of tweets mentioned each city.
(XLSX)

S8 Table The US city names and the number of each US city name mentioned by Twitter users in Chicago. Column A is a list of US city names mentioned by Twitter users living in Chicago. Column B represents state code where the respective city in column A belongs. Column C represents state name where the respective city in column A belongs. Column D and E represent coordinate of each city. F column shows the population in each city. G column represents the number of tweets mentioned each city.
(XLSX)

S9 Table The US city names and the number of each US city name mentioned by Twitter users in Boston. Column A is a list of US city names mentioned by Twitter users living in Boston. Column B represents state code where the respective city in column A belongs. Column C represents state name where the respective city in column A belongs. Column D and E represent coordinate of each city. F column shows the population in each city. G column represents the number of tweets mentioned each city.
(XLSX)

S10 Table The US city names and the number of each US city name mentioned by Twitter users in Charlotte. Column A is a list of US city names mentioned by Twitter users living in Charlotte. Column B represents state code where the respective city in column A belongs. Column C represents state name where the respective city in column A belongs. Column D and E represent coordinate of each city. F column shows the population in each city. G column represents the number of tweets mentioned each city.
(XLSX)

S11 Table The US city names and the number of each US city name mentioned by Twitter users in Houston. Column A is a list of US city names mentioned by Twitter users living in Houston. Column B represents state code where the respective city in column A belongs. Column C represents state name where the respective city in column A belongs. Column D and E represent coordinate of each city. F column shows the population in each city. G column represents the number of tweets mentioned each city.
(XLSX)
